# Unpacking determinants and consequences of food insecurity for insulin resistance among people living with HIV: Conceptual framework and protocol for the NOURISH-OK study

**DOI:** 10.3389/fcdhc.2022.947552

**Published:** 2022-08-16

**Authors:** Marianna S. Wetherill, Casey Bakhsh, Lacey Caywood, Mary B. Williams, Micah L. Hartwell, Denna L. Wheeler, Randolph D. Hubach, T. Kent Teague, Gerwald Köhler, James R. Hebert, Sheri D. Weiser

**Affiliations:** ^1^ Department of Health Promotion Sciences, Hudson College of Public Health, University of Oklahoma Tulsa Schusterman Center, Tulsa, OK, United States; ^2^ Department of Family and Community Medicine, University of Oklahoma School of Community Medicine, Tulsa, OK, United States; ^3^ Tulsa CARES, Tulsa, OK, United States; ^4^ Department of Biostatistics and Epidemiology, Hudson College of Public Health, University of Oklahoma Tulsa Schusterman Center, Tulsa, OK, United States; ^5^ Department of Psychiatry, Oklahoma State University Center for Health Sciences, Tulsa, OK, United States; ^6^ Center for Rural Health, Oklahoma State University Center for Health Sciences, Tulsa, OK, United States; ^7^ Department of Public Health, Purdue University, West Lafayette, IN, United States; ^8^ Department of Surgery, University of Oklahoma School of Community Medicine, Tulsa, OK, United States; ^9^ Department of Psychiatry, University of Oklahoma School of Community Medicine, Tulsa, OK, United States; ^10^ Department of Biochemistry and Microbiology, Oklahoma State University Center for Health Sciences, Tulsa, OK, United States; ^11^ Department of Epidemiology and Biostatistics, University of South Carolina, Columbia, SC, United States; ^12^ Division of HIV, Infectious Disease and Global Medicine, Department of Medicine, University of California, San Francisco (UCSF), San Francisco, CA, United States

**Keywords:** food insecurity, food access, insulin resistance, HIV- human immunodeficiency virus, inflammation, microbiome, community-based participatory research (CBPR), structural equation modeling

## Abstract

**Background:**

Over the past four decades, advances in HIV treatment have contributed to a longer life expectancy for people living with HIV (PLWH). With these gains, the prevention and management of chronic co-morbidities, such as diabetes, are now central medical care goals for this population. In the United States, food insecurity disproportionately impacts PLWH and may play a role in the development of insulin resistance through direct and indirect pathways. The Nutrition to Optimize, Understand, and Restore Insulin Sensitivity in HIV for Oklahoma (NOURISH-OK) will use a novel, multi-level, integrated framework to explore how food insecurity contributes to insulin resistance among PLWH. Specifically, it will explore how food insecurity may operate as an intermediary risk factor for insulin resistance, including potential linkages between upstream determinants of health and downstream consequences of poor diet, other behavioral risk factors, and chronic inflammation.

**Methods/design:**

This paper summarizes the protocol for the first aim of the NOURISH-OK study, which involves purposeful cross-sectional sampling of PLWH (n=500) across four levels of food insecurity to test our conceptual framework. Developed in collaboration with community stakeholders, this initial phase involves the collection of anthropometrics, fasting blood samples, non-blood biomarkers, 24-hour food recall to estimate the Dietary Inflammatory Index (DII^®^) score, and survey data. A 1-month, prospective observational sub-study (total n=100; n=25 for each food security group) involves weekly 24-hour food recalls and stool samples to identify temporal associations between food insecurity, diet, and gut microbiome composition. Using structural equation modeling, we will explore how upstream risk factors, including early life events, current discrimination, and community food access, may influence food insecurity and its potential downstream impacts, including diet, other lifestyle risk behaviors, and chronic inflammation, with insulin resistance as the ultimate outcome variable. Findings from these analyses of observational data will inform the subsequent study aims, which involve qualitative exploration of significant pathways, followed by development and testing of a low-DII^®^ food as medicine intervention to reverse insulin resistance among PLWH (ClinicalTrials.gov Identifier: NCT05208671).

**Discussion:**

The NOURISH-OK study will address important research gaps to inform the development of food as medicine interventions to support healthy aging for PLWH.

## Introduction

The fourth decade of the HIV epidemic marks many milestones in the treatment and management of HIV as a chronic disease. These medical advances have paved the way for new questions about how to optimize the health span for people living with HIV (PLWH), including chronic co-morbidity risk reduction and management. PLWH experience higher rates of diabetes compared to the general US population (1), with one in ten PLWH having diabetes (1) and another three in ten PLWH having prediabetes (2). The underlying insulin resistance that leads to diabetes is also associated with other co-morbidities of concern for PLWH, such as non-alcoholic fatty liver disease (3) and cognitive decline (4). Insulin resistance occurs when a person’s tissues become resistant to insulin-induced glucose uptake (5) and its causes among PLWH are likely multifactorial (5). Non-modifiable risk factors include age and family history, as well as factors specific to HIV, such as certain antiretroviral medications (6, 7) and possibly HIV infection itself (1). Modifiable risk factors for insulin resistance include central adiposity, physical inactivity, and poor diet. Although intervention data are lacking for PLWH, insulin resistance can often be reversed in the general population through healthy eating and other lifestyle behavior changes (6).

### Unpacking the upstream: Contextualizing food insecurity as a risk factor for insulin resistance

While identification of modifiable risk behaviors for insulin resistance among PLWH is an important clinical question, it is unlikely to result in risk reduction unless the context of these behaviors is elucidated and addressed through additional resources and supports. Among PLWH, those with limited financial resources are disproportionately insulin resistant (8). Food insecurity, the uncertain or limited household availability of a healthy food supply, may provide insightful context for understanding predisposition to insulin resistance among low-income PLWH. Food insecurity disproportionately affects PLWH and is a well-established predictor of HIV risk, transmission, and treatment outcomes that operates through nutritional and non-nutritional pathways (9). In the general population, food insecurity is associated with insulin resistance and diabetes, as well as modifiable risk factors for insulin resistance, including inflammatory diets (10), inadequate sleep (11), and physical inactivity (12).

Since food insecurity is a multidimensional, graded phenomenon of increasing severity, each dimension may have distinct influence on health behaviors and outcomes. Therefore, its influence may be understood best when the construct is treated as an ordinal or continuous variable rather than a dichotomous one. For example, marginal and low food security may contribute to insulin resistance *via* overnutrition through the consumption of a pro-inflammatory diet, consisting of low-cost, energy-dense foods with low fiber and micronutrient content (10, 13). In contrast, very low food security can result in undernutrition due to physical hunger and unintentional weight loss. Very low food security may contribute to insulin resistance through other changes in body composition, such as loss of lean muscle mass and possibly functional and morphologic changes in the intestine including intestinal inflammation and gut microbiome alterations (14, 15). Periods of binge eating when food becomes available may contribute to obesity (16, 17). The psychological worry that accompanies food insecurity may influence other behavioral risk factors for insulin resistance, such as tobacco use (18), inadequate sleep (11), and chronic stress (19), while inadequate food intake can limit energy available to engage in physical activity (12) and may encourage tobacco use as a strategy to cope with hunger pangs. Thus, interventions to support long-term food security for PLWH may substantially reduce risk for insulin resistance through many behavioral pathways other than nutrition.

**Figure 1:**
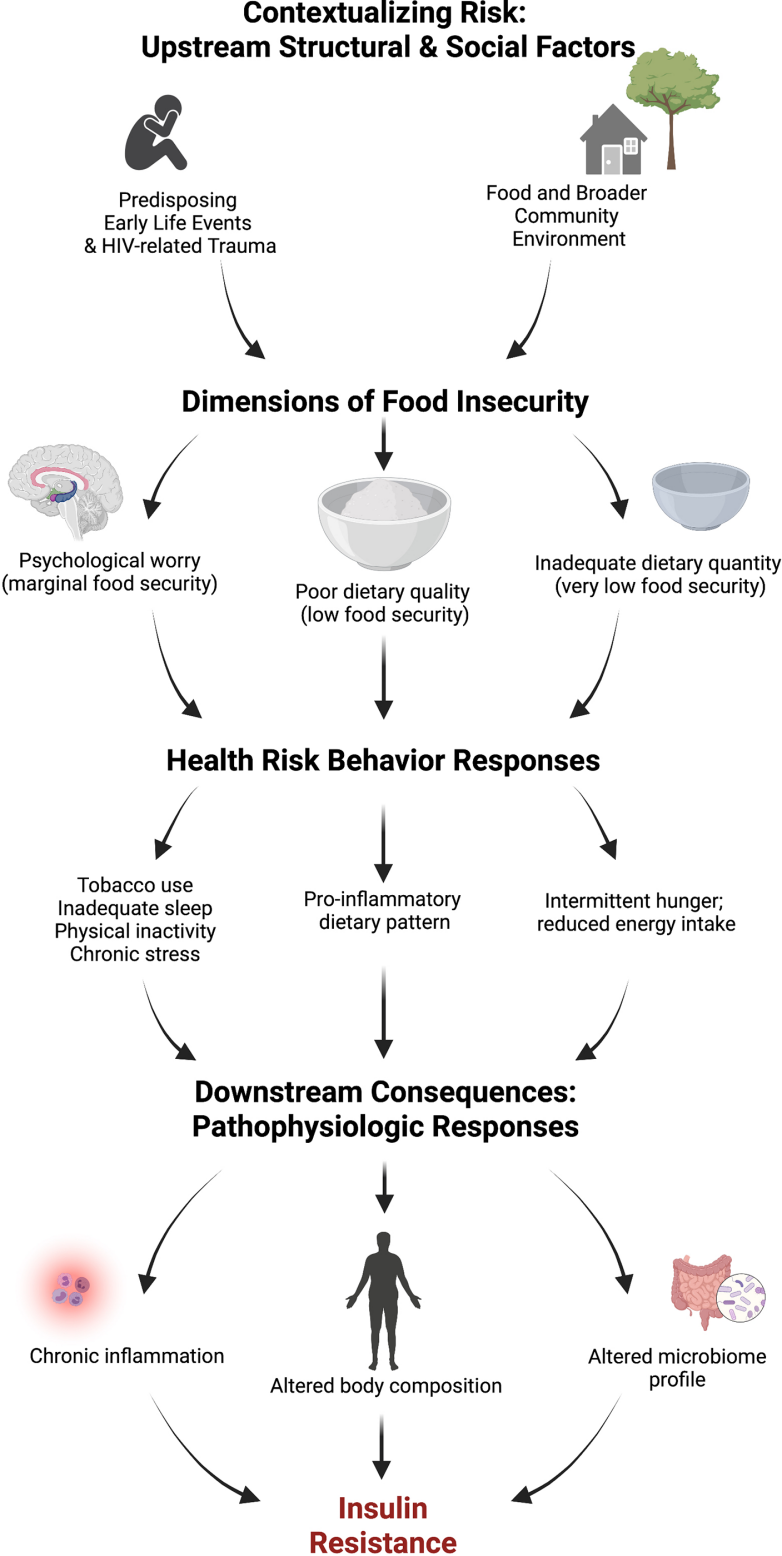
Conceptual framework for food insecurity and insulin resistance. Created with BioRender.com.

Moving further upstream, food insecurity can be viewed as a symptom of broader structural forces that indirectly predispose PLWH towards insulin resistance. Unpacking these determinants can enhance the value of social policies, housing programs, and integrated mental health care efforts as necessary investments for enhancing the metabolic health of PLWH. These forces may include community factors (e.g., residential area deprivation and the local food environment), as well as life course events (e.g., early childhood adversity and trauma related to HIV diagnosis), which may each serve as important effect moderators for food insecurity, health risk behaviors, and biologic pathways. For example, adverse childhood experiences (ACEs) (20), such as physical neglect, are important determinants of food insecurity risk in adulthood (21, 22) and disproportionately reported by PLWH (23). ACEs are associated with epigenetic changes (24) as well as many health risk behaviors (25), which can result in biologic consequences, including chronic inflammation (26), insulin resistance (24), and diabetes risk (27, 28) in the general adult population. Therefore, the influence of these upstream risk factors as potential root causes of chronic co-morbidity risk among PLWH are undoubtedly complex, and their impact likely profound (**Figure 1**).

### Diving downstream: Food insecurity as a potential driver of chronic inflammation among PLWH

At a physiologic, downstream level, additional questions exist for how food insecurity gets “under the skin” to produce pathophysiologic responses associated with insulin resistance. One plausible biological pathway linking upstream forces, food insecurity, and its associated risk behaviors to insulin resistance may be through chronic inflammation. Elevated systemic levels of the inflammatory marker C-reactive protein (CRP) have been linked to food insecurity in the general population (29). Chronic inflammation may be an important contributor to dysglycemia (30), diabetes incidence (31, 32), and other chronic co-morbidities (33) among PLWH, who have higher levels of CRP than the general population (33). Measures of body composition, including body fat and muscle mass, are important biological variable predictors of chronic inflammation (34, 35) and insulin resistance (6, 7, 36, 37). Furthermore, HIV lipodystrophy, body composition changes that are characterized by loss of peripheral fat and deposition of visceral abdominal fat, is also linked to higher fasting insulin levels (38). Central adiposity (independent of BMI) (37) and lipodystrophy (38) in PLWH may directly cause insulin resistance due to accumulation of macrophages in adipose tissue, which secrete proinflammatory mediators (e.g., CRP) that impair glucose uptake into cells (39). Among men with HIV, the residual systematic inflammation that remains after viral suppression may be related to ongoing macrophage activation (40). Among women with HIV, food insecurity has been linked to elevated markers of chronic inflammation, even when controlling for viral suppression and CD4+ T cell count (41).

Additionally, the dietary patterns and periods of prolonged fasting caused by food insecurity may influence the gut microbiome (14), which is increasingly recognized for its role in systemic inflammation and insulin resistance within the general population (42, 43). Thus, food insecurity may exacerbate the consequences of HIV, since infection already disrupts gut homeostasis by reducing CD4+ Th17 and Treg cells in gut-associated lymphoid tissue, which increases intestinal permeability and microbial translocation (44), and drives immune activation and inflammation (45). AIDS history may be particularly detrimental to gut health (44), inflammation (33), and insulin resistance (33). HIV-related alterations in gut microbiome composition, dysbiosis, includes increases in pro-inflammatory *Prevotella* strains, and decreases in anti-inflammatory *Bacteroides* (33). A recent study linked a specific gut microbiome signature with metabolic dysfunction in PLWH, but lacked dietary data (46). Of the few studies that explore the influence of food insecurity or malnutrition on the gut microbiome and its metabolites (14, 47), none have included PLWH.

### Implications for nutrition equity

Malnutrition played a significant role in HIV morbidity and mortality risk in the pre-ART era (48). Community responses to address malnutrition during the early HIV epidemic resulted in the formation of home-delivered meals, other grocery assistance programs, and medical nutrition therapy that typically emphasized intake of high-calorie foods. Over time, many of these programs and services have become institutionalized cornerstones of HIV care across the US. For example, the Ryan White program serves >500,000 unduplicated PLWH nationally (49) and food assistance is an authorized expenditure for Ryan White HIV/AIDS Part A (medical) and Part B (social service) Programs (50).

In contrast with the well-recognized role of food in the prevention and treatment of malnutrition, less is known about how food may be used to reduce chronic co-morbidity risks among PLWH in the modern ART era. Anti-inflammatory dietary patterns may be particularly beneficial for several reasons. Specifically, the Dietary Inflammatory Index (DII^®^), a dietary quality measure that quantifies the inflammatory potential of diet (51), is a useful predictor of chronic inflammation (52), insulin resistance (53–55), and metabolic syndrome (53). Additionally, a high-DII^®^ dietary pattern is associated with food insecurity among lower-income adults in the general population (10). A low-DII dietary pattern is achieved through consumption of wide variety of colorful plant-based foods (fruits, vegetables, nuts, seeds, whole grains, and beans), herbs and spices, and other minimally processed foods. The relationships between food insecurity, inflammatory potential of diet, and insulin resistance among PLWH are yet to be established. An aging cohort of long-term survivors, along with younger generations of more recently diagnosed PLWH, can benefit from HIV-specific nutrition guidance for the prevention of diabetes and related co-morbidities. Research exploring the role of dietary patterns in insulin resistance among PLWH may also be of interest to federal, state, and community stakeholders responsible for management of food assistance programs.

### Objectives

The Nutrition to Optimize, Understand, and Restore Insulin Sensitivity in HIV for Oklahoma (NOURISH-OK) study will result in an integrated, multi-level understanding of how food insecurity contributes to insulin resistance, which is central to the pathogenesis of many chronic co-morbidities in HIV. Its objectives are to: 1) identify the upstream determinants and downstream impacts of food insecurity on pathophysiological processes that may shorten the health span of PLWH, and 2) develop and test a community-driven, science-informed food as medicine intervention to reduce chronic co-morbidity risk among PLWH by addressing key mechanisms in the causal pathway of insulin resistance. We hypothesize that food insecurity will be significantly associated with insulin resistance and that this relationship will be partly mediated by nutritional and non-nutritional health behaviors that increase risk for chronic inflammation. We further hypothesize that these behavioral and inflammatory mediators will significantly differ across all four levels of foods security.

## Methods and Analysis

### Overview of study setting and design

The NOURISH-OK study is being conducted in Oklahoma, a state that ranks among the highest in the nation for adverse childhood experiences (ACEs) (56) and where 54 of its 77 counties are classified as food deserts with all but one county having areas of low food access (57). Among its general adult population, the food insecurity rate (15.6%) exceeds the US rate (11.7%) (58). Its rates of tobacco use, poor diet, and physical inactivity are higher than the national average (59) and the state ranks 2^nd^ highest for obesity, 6^th^ highest for premature mortality (59), and 7^th^ highest for diabetes (59). A recent federal report recognized Oklahoma as being among the top seven states experiencing a high rural HIV burden (60).

The NOURISH-OK study is a 5-year community-based participatory research project that is comprised of three aims. The first aim, initiated in June 2021 with an anticipated enrollment timeline of 18 months, involves a cross-sectional study (n=500) of objective and survey measures to test and refine our conceptual framework of food insecurity and insulin resistance among PLWH. Findings from these analyses will inform the second and third aims of the study, which involve qualitative exploration of significant pathways identified in Aim 1, followed by development and testing of a “food as medicine” intervention to reverse insulin resistance among PLWH that has been prospectively registered (ClinicalTrials.gov Identifier: NCT05208671). Here, we describe the protocol for the first aim of NOURISH-OK.

### Participatory study planning

We developed the NOURISH-OK study using a community-based participatory research (CBPR) approach. This orientation helps to ensure the perspectives and priorities of the study population and other stakeholders are represented throughout the study design, execution, data analysis, and findings dissemination (61). The study’s main community partner, Tulsa CARES, is the state’s largest HIV social services provider and the only dedicated provider of nutrition services for PLWH in northeastern Oklahoma. Founded in 1991, Tulsa CARES is one of four state recipients of Ryan White funding and serves nearly 800 PLWH annually residing in 7 urban counties and 16 rural counties of northeastern Oklahoma.

Prior to the study’s enrollment launch, the main principal investigator (MSW) and community site principal investigator (CB) conducted a series of individual and small group interviews with HIV community stakeholders (n=18) between September 2020-January 2021. Stakeholders included PLWH and those representing public health, rural health, research, Ryan White medical and social services, advocacy, prevention, and outreach sectors. Interviews yielded information on perceived food needs as they relate to well-being for PLWH and community interest in research collaboration to study and address food insecurity. Additionally, the community site principal investigator recruited the initial members of the study’s participant advisory committee (PAC) in January 2021. PAC members provided input on the study logo, frequently asked questions for the study website, recruitment strategies, structure of study incentives, survey items, and enrollment procedures. Finally, Tulsa CARES staff participated in beta testing of participant surveys to provide additional feedback on survey item clarity and flow in March 2021. The University of Oklahoma Health Sciences Center Institutional Review Board (IRB) provided human subjects research approval for all methods described in this protocol. Additional safety protocols pertaining to collection, handling, and processing of saliva, blood, and stool specimens were approved by the University of Oklahoma Health Sciences Center Institutional Biosafety Committee.

### Study population and eligibility criteria

The target population for the NOURISH-OK study is PLWH who are eligible for Ryan White-funded programs and reside in Oklahoma. An estimated 3,000 PLWH access one or more Ryan White-funded programs annually (49) and represent the study’s known recruitment pool. Nearly half (47%) of these individuals identify as a racial or ethnic minority, including 23.8% Black, 5.8% American Indian, and 10.1% Hispanic (49). An estimated 79.7% are male with most Ryan White clients aged 25-44 (42.7%) and 45-64 years (49.2%) (49). Study eligibility criteria require participants be HIV-positive residents of Oklahoma, financially eligible for Ryan White services (i.e., household income <400% federal poverty level), prescribed antiretroviral therapy (ART) for at least the past 6 months, and ability to provide a 10-hour fasting blood sample. Study exclusion criteria includes receiving treatment for cancer or end-stage renal disease.

**Figure 2:**
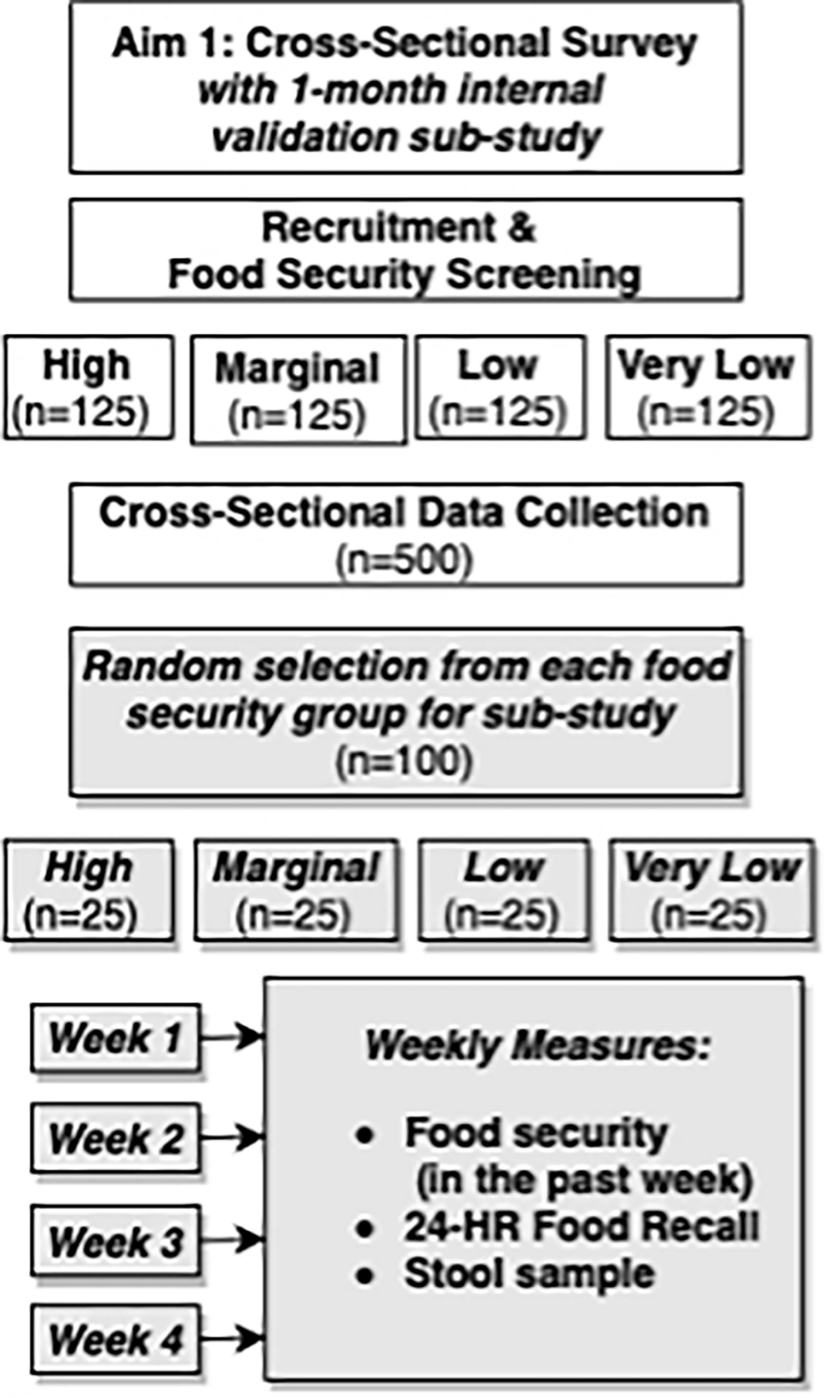
Study design overview for NOURISH-OK Aim 1, which includes a cross-sectional main study and prospective, observational sub-study.

A preliminary food needs study among Oklahoma Ryan White clients in 2012 found a high prevalence of very low (47.6%) and low (19.5%) food security in this population (62). To test our theoretical model of food insecurity as a central mediator linking upstream and downstream determinants of insulin resistance, representation at each level of food security status is needed. Therefore, selection criteria will be based on a food security pre-screening using the 10-item US Adult Food Security Survey Measure (63). Enrollment goals are based on food security status to ensure equal representation across four categories of food security: high (n=125), marginal (n=125), low (n=125), and very low (n=125) (**Figure 2**).

### Main study procedures

#### Recruitment, screening, enrollment, consent and compensation

Our multi-method recruitment strategy will include direct recruitment of Tulsa CARES clients (anticipated reach n=800) through a combination of dear client letters, direct phone calls, and in-person recruitment during Tulsa CARES appointments and sponsored events. Additional dear client letters will be directly mailed by the Oklahoma State Department of Health to individuals participating in the HIV Drug Assistance Program. Indirect recruitment strategies to the broader community of eligible PLWH will include recruitment flyers and business cards at community-based providers, medical clinics serving PLWH, study information booths at community events, and select pharmacies that dispense higher volumes of HIV prescriptions throughout northeastern Oklahoma.

Individuals expressing interest in the study will undergo a telephone or in-person screening interview to confirm eligibility and to discuss study requirements and procedures. Participants will be referred to the study website and encouraged to review its “frequently asked questions” section and informed consent paperwork before their scheduled enrollment appointment. Participants can elect to receive customized appointment reminder texts *via* the HIPAA-compliant Apptoto messaging service and/or phone calls the day before and the day of their scheduled appointment.

The first portion of the appointment will be dedicated to participant completion of study enrollment forms including written informed consent, authorization for use of protected health information in research, and the option to biobank blood samples for use in future studies.

Study compensation was informed through input from PAC stakeholders and will include a $50 gift card from a select list of stores (e.g., Amazon, Wal-Mart, Target), a personalized nutrition assessment results report, and choice of a non-monetary gift valued at $10 (e.g., portable phone battery charger, adult coloring book, paper towel and toilet paper pack). A $15 transportation voucher will additionally be provided to participants living outside of the enrollment site’s county (Tulsa County).

#### Study flow and measures

Study measures will be collected through a combination of interviewer and self-administered surveys, a nutrition and vital signs assessment, and a fasting blood sample. Participants will be encouraged to collect all study measures in a single 2-hour visit. If two sessions are needed, the self-administered survey portion can be completed at a separate appointment within 5 days from the blood sample and nutrition and vital signs assessment. Study measures are summarized in **Tables 1**, **2**.

**Table 1:** NOURISH-OK Aim 1 Study Demographics, Recruitment, and Health Measures.

Variables	Selected Measure
***Demographics***	Household size and composition, household income, race/ethnicity, age, sex (biologic variable); sexual orientation (64) and gender identity (65) as recommended by the Fenway Institute.
***Recruitment strategy***	Recruitment methods reported by participant including all direct and indirect recruitment strategies
***Health-related***	
Heart rate	Digital automatic blood pressure monitor (Medline)
Oxygen saturation	Pulse oximeter (AccuMed)
Blood pressure	Digital automatic blood pressure monitor (Medline)
HIV history	HIV and AIDS diagnosis dates (chart review); Most recent CD4+ and viral load (chart review)
Co-morbidities	**Physical Health Diagnoses:** Self-reported Hepatitis C, Non-alcoholic fatty liver disease, Pre-diabetes, Diabetes, Renal insufficiency or failure, Stroke, Cardiovascular disease, hypertension (adapted from Behavioral Risk Factor Surveillance Survey 2017) (66). **Mental Health:** Depression (Patient Health Questionnaire-9 (PHQ-9)) (67); Anxiety (Generalized Anxiety Disorder-7 (GAD-7)) (68); Cognitive Function 4-item subscale (MOS-HIV) (69) **Chronic Pain:** 2-item subscale (MOS SF-36) (70)
Medications	HIV antiretroviral therapy (ART); insulin secretagogues; insulin; antibiotics; vitamin or mineral supplements (Combination of chart review and self-reported)
Immunization	COVID-19 vaccination status (self-report)
ART adherence	HIV medication adherence (Self-Rating Scale Item (SRSI) (71)

**Table 2:** NOURISH-OK Study Measures.

Variable	Measure(s) or Method(s)
***Upstream Structural and Social Factors***	
Adverse childhood events and protective factors	**Childhood emotional, physical, and sexual abuse:** 10-item survey (Adverse Childhood Events (ACE) survey) (20) **Protective Childhood Experiences:** 7-item survey (72)
HIV diagnosis trauma	**HIV diagnosis as a traumatic stressor:** 2-item survey (73)
Current discrimination	**Expanded Everyday Discrimination Scale:** 10-item survey (74)
Food environment	**Perceived local availability and affordability of fruits and vegetables:** 4-item subscale (Nutrition Environment Measures Survey-Perceived (NEMS-P) (75) **Objective local food access:** Presence or absence of food desert using participant home address (USDA Food Access Research Atlas) (76)
Residential environment	**Neighborhood disadvantage:** Calculated to measure contextual effect of place using participant home address; includes income, education, employment, and housing quality domains to estimate degree of neighborhood disadvantage (Area Deprivation Index) (77) **Rurality:** Continuous measure of rurality scaled from 0 (most urban) to 1 (most rural) based on population size and density, % urban residents, and distance to closest metropolitan area (Index of Relative Rurality) (78, 79)
Social support	**Family & friend support for heart healthy eating habits scales (** 80 **):** including 3 of 9 adapted survey items related to healthy eating (81)
***Food Security***	
Food security	**Food security:** 10-item measure for high (0 pts), marginal (1-2 pts), low (3-5 pts), very low (6-10 pts) food security (USDA Adult Food Security Survey Module) (63)
Food utilization	**Cooking literacy:** 14-item Cooking Skills Confidence Measure (82) **Food literacy:** 19-item Food Skills Confidence Measure (82)
***Health Behaviors***	
Dietary intake	**Dietary quantity:** Absolute and energy-adjusted estimates of energy, macro- and micronutrients, caffeine, and alcohol (Single day 24-hour food recall using ASA24^®^) **Dietary quality:** Inflammatory potential of overall diet (DII^®^/E-DII^®^ using ASA24^®^data) **Skin carotenoid concentration biomarker:** Continuous measure of usual carotenoid and fruit/vegetable intake (Reflections Spectroscopy (RS) Veggie Meter^®^ device) (83) **Mealtime habits:** Meals/day; calories/meal (ASA24^®^); eating meals with others
Sleep quality and quantity	**Sleep quality and quantity:** 3-item measure of sleep duration, latency, and complaints (2007 NHANES National Center for Health Statistics Sleep Disorders survey) (11)
Physical activity	**Physical activity:** 3-item aerobic activity summary index (PA-I) (84) **Sedentary behavior:** 2-item measure to estimate sitting behavior hours per day (adapted from International Sedentary Assessment Tool) (85) **Estimated cardiorespiratory fitness:** Validated gender-specific formula using age, waist circumference, height, weight, resting heart rate, and self-reported physical activity (84, 86)
Tobacco use	**Self-report tobacco use:** 2-item measure for never, ever, or current smoker classification (87); 1-item measure for smokeless tobacco use; 1-item measure for other forms of nicotine **Nicotine dependence:** 2-item measure (88) **Objective first- and second-hand tobacco exposure:** Cotinine (Genway Biotech - Cotinine Direct ELISA) (89)
Alcohol use	**Alcohol use and abuse**: 3-item survey (AUDIT-C) (90) **Alcohol intake past 24-hours:** ASA24^®^ (91)
Substance use	**Substance use:** 2-items from 8-item NIDA-Modified ASSIST Version 2.0 to assess lifetime and past 3 month medical and nonmedical substance use for 9 categories of substances **Medical marijuana card status:** self-report
Chronic stress	**Perceived stress:** 10-item survey (PSS-10) (92) and 1-item COVID-19 pandemic stress (93)
***Downstream Pathology***	
Chronic inflammation	**High sensitivity C-reactive protein** (MSD - Vplex CRP Kit)
Body composition and malnutrition risk indicators	**Wrist circumference:** Measured in centimeters using Singer tape measure to estimate body frame size (ADAM Medical Encyclopedia) (94) **Grip strength:** Measured using dynamometer (Jamar) as screening indicator for malnutrition (95) **Waist circumference:** Measured in centimeters (Adapted from NHANES 2019-2020 Procedures) (96) **Segmental BIA:** Fat, lean dry mass, and phase angle estimates for truncal, right arm, left arm, right leg, left leg, total body (RJL Systems Quantum V Segmental BIA) (97) **Height and weight:** SECA stadiometer Model #220, SECA Electronic Scale Model #703 **Body mass index:** kg/m^2^
Glucose homeostasis	**Impaired fasting glucose:** Defined as fasting plasma glucose between 100-125 gm/dL (98) **Fasting glucose** (MyBioSource – Glucose ELISA) **Fasting insulin** (MSD - Vplex insulin Kit) **HgbA1c** (A1cNOW^®^ PTS Diagnostics)
Insulin resistance (primary outcome)	**Insulin resistance:** Quantitative Insulin Sensitivity Check Index “QUICKI” = 1/[log(I_0_) + log(G_0_)], where I_0_ is fasting insulin (microunits per milliliter) and G_0_ is fasting glucose (milligrams per decaliter) (99)

#### Blood sampling, processing, and analysis

Before blood samples are collected, research staff will confirm each participant has fasted a minimum of 10 hours by asking the last time the participant ate or drank anything besides water. Venous blood samples will be collected by a trained phlebotomist using one BD Vacutainer serum tube, one BD Vacutainer EDTA plasma tube, one BD Vacutainer Heparin tube, one BD Vacutainer P800 plasma tube, and one BD Vacutainer CPT tube for peripheral blood mononuclear cells (PBMC). Samples will be transported to the designated study lab and processed within 2 hours of draw according to manufacturer’s instructions. Plasma and serum aliquots will be stored at −80°C until analysis. Peripheral blood mononuclear cells aliquots will be stored for less than 75 hours at −80°C and then transferred to liquid nitrogen vapor phase for cryopreservation until analysis. Laboratory measures, including serum insulin, blood glucose, c-reactive protein, hemoglobin A1c, and blood cotinine, are summarized in **Table 2**.

#### Nutrition and vital signs assessment

Trained research assistants will then conduct a nutrition assessment including height, weight, waist circumference, wrist circumference, grip strength, and a segmental bioelectrical impedance analysis test. Additional vital signs will include resting heart rate, blood oxygen level, and blood pressure. Resting heart rate, waist circumference, gender, and age will be used to estimate cardiorespiratory fitness (84, 86). We will collect one 24-hr food recall using the Automated Self-Administered 24-hour (ASA24^®^) Dietary Assessment Tool as our primary measure of dietary intake from all subjects (91). We will additionally measure skin carotenoid concentration using Reflections Spectroscopy (RS) (83). This will serve as a concentration biomarker to adjust for measurement error in estimated carotenoid and total fruit and vegetable intake from the ASA24^®^-generated dietary intake estimates.

We will measure height and weight (without shoes) to calculate body mass index (BMI) using a medical grade stadiometer and scale. We will measure central adiposity using waist circumference adapted from NHANES guidelines (96). To minimize participant discomfort, participants will identify body landmarks for self-measure in front of a mirror with assistance from staff. We will conduct segmental bioelectrical impedance analysis (BIA) using the Quantum V Segmental BIA (RJL Systems). Participants who may be pregnant or who have implanted electronic devices will be excluded from this measure. Participants with metal surgical implants present on one side of the body will complete a one-sided measure using the side of the body without the implant. Segmental body fat readings (right arm, left arm, right leg, left leg, and torso) will be used to further identify body type pattern associated with insulin resistance (97). We will minimize BIA measurement error associated with dehydration by providing 16 oz. of water 30 minutes prior to measurement. Collectively, these measures will be used to estimate nutritional and functional status.

#### Chart review and surveys

After receiving authorization from participants through the informed consent process, research staff will conduct a medical chart review to obtain participant’s most recent CD4+ T cell and viral load measures and HIV and AIDS diagnosis dates. Alternatively, participants will be required to provide proof of HIV positivity and a copy of their most recent lab work in the past 6-12 months if this information is not accessible *via* chart review. On the day of study enrollment, participants will also be asked to confirm their residential address, demographics, and medication lists from their existing chart records before this information is added to the study database. Residential address will be used to create objective measures of the community environment, including food desert status (100) and residential area socioeconomic deprivation (77).

A 233-item self-administered survey will collect remaining study measures. These measures include early life events, trauma related to HIV diagnosis, perceived availability of food in the participant’s home and neighborhood, nutrition and health behaviors, food and cooking literacy, social support, and emotional, mental, and physical health history.

#### Exit interview and participant follow-up calls

At the end of their study visit, each participant will receive an information packet that includes a list of community resources, including food assistance and mental health services, contact information for study staff, and several study business cards to share with others who may be eligible for the study. Any participant who reported suicidal ideations while completing the PHQ-9 will be connected on-site with a licensed clinical social worker for further evaluation and follow up at this time. Several days after the study appointment, a designated member of the research team will attempt to contact participants *via* phone to collect qualitative feedback for quality improvement purposes. Feedback areas include satisfaction and opportunities for improvement with research staff, study procedures, incentives, and other general feedback.

### Prospective observational sub-study procedures

#### Replicate dietary intake and food security recalls

Upon completion of the cross-sectional study, participants will be randomly selected within their food security group for an invitation to participate in a 4-week observational sub-study. Elucidating the influence of food insecurity on dietary intake presents an important methodologic challenge because intake estimates from those with low and/or very low food security may potentially have higher error as compared to those with high or marginal food security. These differences in error may be due to wider fluctuations in food access throughout each month (101) and seasonally (102) for food insecure groups. Therefore, the primary purpose of the sub-study is to collect replicate dietary recalls to obtain a more precise estimate of usual dietary intake for each food security group, in addition to exploring how food security fluctuates from week-to-week. Trained interviewers will collect replicate dietary recalls and food security surveys *via* phone. At sub-study enrollment, subjects will receive a printed guide illustrating standard food units of measure for reference during phone calls to improve reporting accuracy.

#### Exploratory microbiome measures

Sub-study participants will also be asked to provide a single saliva sample and 4 weekly stool samples for exploratory analyses of microbiome profiles and variables of interest. At the time of sub-study enrollment, participants will provide their saliva sample using a self-sampling saliva test kit (OMNIgene*ORAL, OM-501; DNA Genotek, Ottawa, Ontario, Canada) that allows for DNA stability at room temperature for up to 1 year. Participants will receive four stool collection kits (OMNIgene*Gut, OMR-200.100; DNA Genotek) that will be marked with unique identifiers for self-collection at home. Research staff will instruct study subjects on how to use the kits and store stool samples, which can be kept for up to 60 days at room temperature.

On a randomly-selected day each week, participants will receive a text or phone call notification from research staff requesting to complete their weekly phone survey which includes a 24-hour dietary recall followed by collection of a sample from their next stool. Participants will have the option to return kits by mail or by hand to the enrollment site. Participants will receive up to a $100 gift card upon completion of the sub-study ($25 for each week dietary recall is completed and stool sample is collected).

Upon arrival to the research lab, handling and storage of the saliva and stool samples will follow the manufacturer’s recommendations. For precautionary measures, sample processing and DNA isolation procedures will be under Biosafety Level 2 (BSL-2) containment. Samples will be stored under suitable conditions (long-term storage at -80°C) until processing for DNA isolation. A commercial DNA isolation kit (e.g., Zymo Research ZymoBIOMICS DNA Miniprep Kit) will be used to isolate DNA from saliva and fecal sample aliquots. Isolated DNAs will be quality controlled using spectrophotometry for measurement of DNA concentration and purity (260nm/280nm, 260nm/230nm ratios) as well as agarose gel electrophoresis for determination of DNA integrity. If indicated, isolations will be repeated.

Salivary and fecal DNAs will be processed for 16S ribosomal RNA gene-based analyses of the respective microbiotas. We will target the hypervariable region V4 of 16S rRNA genes using dual-indexed, paired-end amplicon sequencing with an Illumina MiSeq system (Illumina, Inc., San Diego, CA) as described by Kozich et al. (103). The resulting sequence reads will be analyzed following state-of-the-art bioinformatics pipelines for quality control, taxonomic profiling, and differential abundance analyses of microbial communities. Sub-study measures are summarized in **Table 3**.

**Table 3:** NOURISH-OK Aim 1 Sub-study Observational Measures.

	*Sub-Study Enrollment Baseline Measures*
**Gastrointestinal history**	**History of gastrointestinal surgery or health conditions** (self-reported) **Mode of birth (C-section or vaginal delivery)** (self-reported) **Frequency and timing of bowel movements** (self-reported) **Microbiome-altering sexual practices** 12-month history (self-reported)
**Oral health history**	**Oral disease or infection** (self-reported) **Food avoidance due to mouth, teeth, or gums:** 2-item question adapted from (104)
**Medication history**	**Microbiome-altering medications**: 3-month history of antibiotics, antifungals, laxatives, proton pump inhibitors, and probiotics use (self-reported) **Medications taken in the last 12 hours** (self-reported)
**Oral microbiome**	**Most recent consumption of food, drink, caffeine, or tobacco** at time of sample (self-reported) **Self-sampling saliva test** (OMNIgene•ORAL, OM-501; DNA Genotek) **Diversity index:** Alpha and beta diversity analyses **Relative abundance:** Taxonomic analyses
	***Weekly Measures (4 timepoints)***
**Dietary intake**	**Dietary quantity:** Absolute and energy-adjusted estimates of energy, macro- and micronutrients, caffeine, and alcohol (Single day 24-hour food recall using ASA24^®^) **Dietary quality:** Inflammatory potential of overall diet (DII^®^/E-DII^®^ using ASA24^®^data)
**Food security**	**Food security in the 30 days:** 10-item USDA Adult Food Security Survey Module (Timepoint 1) (63) **Food security in the past week:** Adapted from previous study to assess daily food security (105) that includes modified HH2, HH4, AD2 measures from 10-item USDA Adult Food Security Survey Module (Timepoints 2 – 4) (63)
**Sleep quantity**	**Sleep quantity:** 1-item measure of sleep duration in the past 24-hours (adapted from 2007 NHANES National Center for Health Statistics Sleep Disorders survey) (11)
**Physical activity**	**Physical activity:** 2-item measure of aerobic activity in the past 24-hours adapted from (84) **Sedentary behavior:** 1-item measure to estimate sedentary behavior minutes in the past 24-hours (adapted from the International Sedentary Assessment Tool) (85)
**Intestinal transit time**	**Self-reported stool sample consistency**: Bristol stool chart (106) **Date and time of most recent bowel movement** at time of dietary food recall (self-report)
**Gut microbiome**	**Self-sampling stool test** (OMNIgene•Gut, OMR-200.100; DNA Genotek) **Diversity indices:** Alpha (e.g., Shannon, Faith’s phylogenetic diversity) and beta diversity analyses **Relative abundance** of specific bacterial groups (e.g., Firmicutes, Bacteroidetes, Proteobacteria, Bacteroides, Prevotella, etc.) **Differential abundance analyses** using participant and dietary metadata
**Medication changes**	Changes in microbiome-altering prescribed and over-the-counter medications reported during Aim 1 enrollment and sub-study participation period

### Data storage and management

To protect confidentiality, each participant will be assigned a unique study identifier that will be used to link study datasets. Identifying information, including participant name, contact information, and the unique study identifier will be stored in a separate database not to be included in the data analysis. Survey data will be collected and managed using REDCap electronic data capture tools (107, 108) that are hosted on a secure server at the University of Oklahoma Health Sciences Center. Biomarker and body composition data will be manually entered into the REDCap database by research staff. De-identified microbiome sample data will be stored and analyzed at Oklahoma State University Center for Health Sciences. Analytic output files (dietary recall data) will be directed downloaded from the ASA24^®^ website and saved on a secure server at the University of Oklahoma Health Sciences Center. Before analysis, these dietary data will be reviewed for missing nutrient data, portion outliers, and nutrient outliers in accordance with the general guidelines from the National Cancer Institute (NCI) regarding how to review and edit ASA24 data.

### Statistical analysis

We will first examine associations between continuous measures, such as food insecurity on the 10-point scale, DII^®^ scores, CRP level, and various measures of glucose homeostasis, using linear regression. We will compare means using t-tests for categorical variables with 2-levels and ANOVA for variables with 3 or more levels, such as the 4-level food security categorical variable (high, marginal, low, very low food secure).

#### Insulin sensitivity

We will use fasting glucose and insulin measures to calculate the quantitative insulin sensitivity check index (QUICKI) as the primary outcome measure for our analyses where a lower value indicates greater insulin resistance (99). Due to its reliability, reproducibility, accuracy, and positive predictive value across a wide range of co-morbidities, the QUICKI is a widely-accepted surrogate index measure of insulin sensitivity that is recommended for larger, community-based research studies when the laborious, clinic-based hyperinsulinemic-euglycemic clamp technique is not feasible (107). The QUICKI uses the empirically-derived equation QUICKI = 1/[log(I_0_) + log(G_0_)], where I_0_ is fasting insulin (μU/ml), and G_0_ is fasting glucose (mg/dl) (99). The QUICKI has been shown to perform equally to or slightly better than the homeostasis model assessment of insulin resistance (HOMA-IR) across the insulin sensitivity-resistance spectrum (108, 109). Additionally, it offers a slight advantage for measuring changes in insulin sensitivity over time (107, 110), which in consideration of our third aim (examining the effects of a food is medicine intervention on insulin resistance), the QUICKI was selected as the most appropriate outcome measure for the NOURISH-OK study.

#### Dietary intake

We will calculate DII^®^ scores (crude and energy-adjusted) developed by Hebert and colleagues (51) for the full sample with measurement error correction using information from the sub-study. The DII^®^ and the energy-adjusted DII (E-DII™) scores are calculated based on dietary intake of food parameters (i.e., a combination of whole foods and nutrients associated with inflammation). Scores can range from a theoretical low of around -9 to a theoretical high of +8; however, scores are rarely outside the range of -6 to +6. Negative scores represent an anti-inflammatory dietary pattern, while positive scores represent a pro-inflammatory dietary pattern. We hypothesize that DII^®^ score will be positively associated with insulin resistance. We will conduct sensitivity analyses of the relationship between DII^®^ scores and insulin resistance at each level of food security. We hypothesize that DII^®^ scores will be more strongly associated with insulin resistance for food insecure groups than food secure groups (111). These analyses will inform a list of candidate intervention foods for further evaluation in Aim 2. Dietary fiber has been inversely associated with BMI in PLWH (112) and is a source of sustenance for beneficial gut bacteria (113), thus we will also compare the impact of dietary fiber on insulin resistance. We will further explore dietary component factor loadings contributing most strongly to the DII^®^ scores and use discriminant analysis to understand the relationship between DII^®^ score and reported patterns of intake.

#### Estimating dietary intake measurement error

For dietary intake estimates, we will conduct an internal regression calibration study using the sub-sample of 100 subjects with four additional dietary recalls to estimate and adjust for measurement error in the remaining sample with single-day recalls (114). We will use the methods of triads to combine dietary recall data with dermal carotenoid scores to more precisely estimate true carotenoid and total fruit and vegetable intake (115, 116). Since chronic inflammation and smoking are both independently associated with lower beta-carotene concentrations (116), we will control for these confounders, if needed. Additionally, we will explore whether measurement error varies by food security status using sensitivity analysis to estimate and adjust for measurement error at each food security level. We will estimate variance in SAS using the Restricted Maximum Likelihood Estimation method and variance ratios as the error variance/variance across individuals. The within- (CVw=Sw/mean of each nutrient) and between-individual (CVb = Sb/mean of each nutrient) coefficient of variation (CV) will be estimated using Beaton’s formula (117). The CVw will then be used to estimate the number of 24-hr food recall days needed to estimate accurate dietary intakes among PLWH, and whether the number of recall days differ by food security status (117). Findings will be applied to modify the number of dietary recalls collected to evaluate the impact of the NOURISH-OK intervention (Aim 3).

#### Validation of self-reported non-nutritional risk behaviors

As described in our conceptual framework, food insecurity may influence risk factors beyond dietary intake, such as tobacco use and stress. Therefore, we will validate self-report tobacco use with serum cotinine, which is recognized by the Centers for Disease Control and Prevention as the preferred biomarker for tobacco use (89). Additionally, we will use regression to assess variance explained in the stress biomarker CRP values by PSS scores. Results from these validation studies will inform selection of appropriate measures to evaluate the impact of the NOURISH-OK intervention on additional health risk behaviors.

#### Final structural equation modeling

Finally, we will test our conceptual model using structural equation modeling, a method that allows simultaneous measurement of the strength and significance of predictor variables *via* direct and indirect pathways on a single outcome established *a priori* by theory (118). The theoretical framework draws from existing conceptual models (16, 119) that position physical and social community environments as risk regulators (119) for household food insecurity and its resulting individual nutritional and non-nutritional risk behaviors (16), which become embodied through pathophysiologic responses (119) (**Figure 1**). Sample size recommendations vary widely for studies that employ structural equation modeling, with lower estimates citing 200 cases for SEM models (118). We have selected a conservative sample size of 500, which exceeds more recent recommendations for up to 460 cases for multi-factor, multi-indicator models (120).

**Figure 3:**
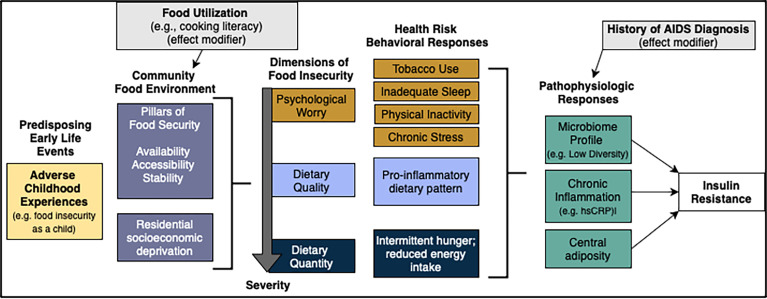
Structural equation model of food insecurity and insulin resistance. Additional co-variate demographics not depicted. Created with draw.io.

The SEM will simultaneously estimate direct and indirect effects of continuous exposure variables in the directional pathway of food insecurity and insulin resistance (**Figure 3**). Specifically, we will test the influence of upstream and social factors on food insecurity, and whether food utilization skills modify the association between community food access and food security. As an intermediate variable in the SEM, food security status will be used to predict insulin resistance using behavioral and pathophysiologic mediators with AIDS diagnosis as a potential effect modifier. We will use similar methods developed in our past research (121), including quasi-maximum likelihood estimation with Satorra-Bentler correction to measure direct and indirect paths between all variables. We will use Chi-square, Root Mean Square Error of Approximation (RMSEA), and Comparative Fit Index (CFI) to assess model fit. We will conduct invariance testing using AIDS status to compare model differences between those with and without AIDS diagnosis history. We will re-specify the model, if needed, using a theory-informed approach. Analyses will be conducted using R, Version 4.0.2.

#### Microbiome exploratory analyses

The highly complex nature of microbiome data has led to a continuing evolution of analyses methods. We will use the next-generation microbiome bioinformatics platform QIIME 2 (122) as the standard analysis package from raw sequences to statistical evaluation of the cross-sectional saliva and the longitudinal (time series) stool microbiota study results. Additional methods such as LEfSe (Linear discriminant analysis Effect Size) (123) will be included in our analysis pipeline.

The compositional and zero-inflated nature of microbial community profiling data requires specialized, preferably non-parametric approaches for statistical analyses of associations with clinical or dietary variables. Due to high rates of periodontal disease in people with FI (124, 125) and PLWH (126, 127), we will explore correlations between oral microbiota profiles (composition and diversity) and insulin resistance, and whether self-reported measures of poor oral health, food insecurity, and chronic inflammation mediate these relationships. Additionally, we will explore whether GMB qualities (i.e., overall GMB diversity index; specific biomarker components, such as *Prevotella*, *Bacteroides* (128)) mediate the association between dietary intake (i.e., DII^®^; individual constituents such as fiber) and insulin resistance. The variance estimates and intraclass correlation coefficients from repeated measures of GMB and dietary recall data will be used to refine data collection and statistical analysis methods for the NOURISH-OK intervention (Aim 3).

## Discussion

The NOURISH-OK study is a novel exploration of how the distinct psychological, dietary quality, and dietary quantity components of food insecurity may influence health risk behaviors and insulin resistance among PLWH. It challenges prevailing food insecurity research paradigms that traditionally emphasize one level of understanding (e.g., behavioral vs. molecular) by simultaneously considering environmental, psychosocial, behavioral, and biological factors in the pathogenesis of insulin resistance, and how food insecurity may amplify or dampen these pathways. Further, its conceptual framework incorporates the role of traumatic events as potential underlying contributors to food insecurity and its associated outcomes. Collectively, the first aim of the NOURISH-OK study is designed to provide HIV researchers, clinicians, and other stakeholders with key leverage point opportunities for building nutrition equity among PLWH, including development of evidence-informed food as medicine programs that address dimension(s) of underlying need.

In addition to elucidating the various potential mechanisms by which food insecurity shapes health risks, the NOURISH-OK study will support validation of dietary assessment measures in PLWH. These measures include dermal carotenoids as a biomarker for total carotenoid and total fruit/vegetable intake and construct validity of the DII^®^ as a predictor of inflammation in PLWH. Because carotenoids, fruits, and vegetables correlate with other nutrients that comprise the DII^®^ score and inflammatory markers, dermal carotenoids may be a useful non-invasive alternative to intensive dietary collection methods in future PLWH studies, including measures of intake and dietary intervention adherence. Although tested extensively in diverse populations (129, 130), this surrogate biomarker has not been studied among PLWH. The NOURISH-OK sub-study will further address methodologic gaps by exploring whether there are differences in dietary intake measurement error based on food insecurity status. If food security status is found to be related to measurement error, it will be paramount that future research investigating the role of food insecurity on dietary intake incorporate methods to adjust for this error. Furthermore, the sub-study’s oral and gut microbiome analyses will answer important questions about whether food insecurity is associated with distinct microbiome profiles and whether these profiles are attributable to diet and insulin resistance among PLWH. Finally, the NOURISH-OK study addresses additional methodologic gaps in health behavior research by including additional validation studies of other lifestyle behaviors among PLWH beyond diet, including self-reported tobacco (validated with serum cotinine) and perceived stress (validated with CRP).

An important limitation of the NOURISH-OK study’s first aim is the inability to establish temporal linkages among variables within the conceptual framework due to its cross-sectional design. Since the relationships between food insecurity and HIV-related outcomes may operate bidirectionally (9), additional research will be necessary to elicit a deeper understanding of complex relationships within the framework and to refine proposed mechanisms. The study’s second aim, comprised of qualitative inquiry with PLWH who experience food insecurity, will help to address this limitation by allowing the research team to triangulate data pertaining to potential directionality and context of significant pathways identified in Aim 1.

In closing, the NOURISH-OK promotes structural equity in HIV research by operating through a community-academic research partnership that directly involves PLWH at the decision-making table (131). These contextual factors will help to ensure current study activities incorporate community concerns and priorities, while simultaneously building capacity to plan and engage in future research that responds to evolving community needs.

## Ethics statement

The studies involving human participants were reviewed and approved by University of Oklahoma Health Sciences Center Institutional Review Board (study #12509). The patients/participants provided their written informed consent to participate in this study.

## Author contributions

MSW, conceptualized and obtained funding the NOURISH-OK study, designed the conceptual framework depicted in **Figure 1**, led development of the methods, and wrote the original manuscript draft; CB, contributed to the overall study design and recruitment strategies, led participant advisory board activities; LC, led project administration activities pertaining to database development, writing of research protocol, participant enrollment, data collection, and monitoring; MBW, led data curation activities pertaining to data management, cleaning and integrity and supervised statistical support staff; MLH led methods for structural equation modeling; RH, advised on recruitment methods for rural Oklahomans living with HIV; RDW, advised on rural sampling and classification methods; TKT, led selection of blood and serum laboratory measures and supervision of laboratory personnel; GK, contributed to the literature review, advised on microbiome sub-study design and oversaw analysis of oral and gut microbiome samples; JDH, co-developed the Dietary Inflammatory Index^®^ and advised on nutrition epidemiologic methods; SDW, advised on study design, participant safety protocols, and survey measures as an HIV food insecurity content expert. All authors reviewed, edited, and approved the final version of the manuscript.

## Funding

Research reported in this publication was supported by National Institute of Diabetes and Digestive and Kidney Diseases of the National Institutes of Health under Award Number R01DK127464 and by the National Institute of General Medical Sciences of the National Institutes of Health under Award Number U54GM104938. The content is solely the responsibility of the authors and does not necessarily represent the official views of the National Institutes of Health.

## Acknowledgments

The authors thank members of the participant advisory committee for their valuable contributions to the NOURISH-OK study methods. The authors also acknowledge the contributions of Brenda Davis and Ashlee Rempel from the laboratory of T. Kent Teague at the University of Oklahoma Integrative Immunology Center for their assistance with details of the study protocol involving sampling, processing, and handling of all blood samples. The authors thank Victoria Thomas, MD, MPH, Raegan Tremblay, BGS, and Jacqueline Dukes, BS, for their assistance with the preliminary development of the NOURISH-OK study procedures manual as well as Donna Fesler, MS, RN, CNEcl, and Kia Beverly for their training and consultation in phlebotomy procedures. We acknowledge the Harold Hamm Diabetes Center for its supportive consultation throughout the development of the NOURISH-OK study.

## Conflict of interest

Unrelated to the present work, MSW previously served as an advisor for Food and Society at the Aspen Institute’s Food is Medicine Initiative and is a consultant for the Sunflower Foundation’s Food is Medicine Initiative.

The remaining authors declare that the research was conducted in the absence of any commercial or financial relationships that could be construed as a potential conflict of interest.

## Publisher’s note

All claims expressed in this article are solely those of the authors and do not necessarily represent those of their affiliated organizations, or those of the publisher, the editors and the reviewers. Any product that may be evaluated in this article, or claim that may be made by its manufacturer, is not guaranteed or endorsed by the publisher.

## Abbreviations

ACEs, Adverse childhood experiences; BMI, Body mass index; CRP, C-reactive protein; DII^®^, Dietary Inflammatory Index; LEfSe, Linear discriminant analysis Effect Size; NOURISH-OK, Nutrition to Optimize, Understand and Restore Insulin Sensitivity in HIV for Oklahoma; PLWH, People living with HIV; PSS, Perceived stress scale; QUICKI, quantitative insulin sensitivity check index; SEM, Structural equation modeling.
